# Clinical factors associated with jawbone remineralization after nonsurgical treatment and imaging-based insights into its processes

**DOI:** 10.1007/s11282-025-00867-6

**Published:** 2025-10-01

**Authors:** Kiichi Shimabukuro, Pongsapak Wongratwanich, Masaru Konishi, Fahri Reza Ramadhan, Megumi Nose, Masahiko Ohtsuka, Toshikazu Nagasaki, Yoshikazu Suei, Takashi Nakamoto, Naoya Kakimoto

**Affiliations:** 1 Department of Oral and Maxillofacial Radiology, Graduate School of Biomedical and Health Sciences, Hiroshima University, 1-2-3 Kasumi, Minami-Ku, Hiroshima City, Hiroshima, 734-8553 Japan; 2 Department of Oral Biomedical Sciences, Division of Oral Diagnosis, Khon Kaen University, Khon Kaen, Thailand; 3 Department of Oral and Maxillofacial Radiology, Hiroshima University Hospital, Hiroshima, Japan

**Keywords:** Remineralization, Nonsurgical treatment, Bone metastases, Bone destruction

## Abstract

**Objectives:**

This study aimed to clarify the clinical factors and processes associated with jawbone remineralization at sites of tumor-induced bone destruction after nonsurgical treatment by analyzing clinical factors, computed tomography (CT) values, and positron emission tomography (PET)/CT images.

**Methods:**

The data of 58 patients with jawbone destruction due to oral tumors or metastatic lesions who received radiation therapy or chemotherapy were retrospectively analyzed. Clinical factors, treatment modalities, and CT and PET/CT images at the time of bone destruction and remineralization were analyzed. CT values were measured at 0, 3, 6, and 12 months after treatment, and the ratios to the reference values were calculated.

**Results:**

Among the 58 patients studied, 23 had remineralization, 25 showed no significant changes, and 10 had progressive bone resorption. Significant factors influencing remineralization included age, tumor histology, site of jawbone destruction, type of bone destruction, and radiation dose. The mean CT values at the remineralized sites increased progressively, and significant age- and sex-related differences were observed at 12 months. Patients younger than 18 years had CT values and bone-morphology changes comparable to reference sites. Post-treatment PET/CT revealed significant ^18^F-FDG accumulation with metabolic activity patterns resembling those of bone remineralization.

**Conclusions:**

Bone remineralization following jawbone destruction is influenced by patient-specific characteristics and treatment-related factors. Changes in CT values during bone remineralization and metabolic activity detected using PET/CT provide insights into the underlying processes of bone remineralization.

**Trial registration:**

Registration number: E2023-0253.

Registration date: February 19, 2024 (Hiroshima University Epidemiological Research Ethics Committee).

## Introduction

The increasing incidence and mortality rate of lip and oral cavity cancer, as reported by the International Agency for Research on Cancer (IARC) Global Cancer Observatory (GLOBOCAN), present a significant global health concern [1, 2]. This alarming trend, exacerbated by the aging population alongside remarkable advancements in cancer treatments and imaging techniques, has resulted in the increasing number of survivors of long-term cancer who have jawbone destruction [3]. Bone invasion of the mandible or maxilla is a commonly observed complication in patients diagnosed with oral squamous cell carcinoma, with nearly half experiencing such issues [4, 5]. Metastases to the oral cavity are relatively rare, accounting for approximately 1–8% of all cancers [6, 7]. However, the jawbone is more frequently affected than oral soft tissues. The mandible is the most involved site within the oral cavity, followed by the gingiva, maxilla, and tongue [6, 8–10]. The jawbone is a common site for bone invasion in oral squamous cell carcinoma. Other cancers more frequently metastasize to different bones, with the vertebrae being the most common destination. Approximately half of patients with osteolytic bone lesions in the vertebrae have remineralization after radiation therapy [11–14]. Several factors, including sex, primary tumor site, histology, radiation dose, treatment planning, and the use of bisphosphonates, have been identified as potential contributors to remineralization [13–19]. Notwithstanding, the prognostic significance of remineralization has not been established, highlighting a crucial gap in our understanding [20–24].

Studies on remineralization of the jawbone are lacking. This oversight may be attributed to several challenges unique to the oral cavity, including the predominance of surgery as the primary treatment modality for oral cancer, risk of radiation-induced osteonecrosis of the jaw, and potential for medication-related osteonecrosis associated with bone-modifying agents. These factors may have contributed to the limited documentation of jawbone remineralization.

The purpose of this study was to evaluate the clinical factors and imaging insights into its processes associated with jawbone remineralization, focusing on patients undergoing radiation therapy or chemotherapy as the primary treatment for cancers involving jawbone destruction. The clarification of these features is expected to provide insight into understanding bone regeneration after nonsurgical treatment and is expected to guide the development of therapeutic strategies to enhance bone healing and improve clinical outcomes.

## Materials and methods

### Study population

The Hiroshima University Epidemiological Research Ethics Committee (registration number: E2023-0253) approved this retrospective study. Informed consent was obtained from the study participants using an opt-out method. The study initially recruited 3,607 patients between 2000 and 2023. These patients had undergone positron emission tomography–computed tomography (PET/CT) or bone scintigraphy at Hiroshima University Hospital and had tracer accumulation within their jawbones. Subsequent reviews of additional medical imaging modalities, including CT and magnetic resonance imaging (MRI), were conducted to identify patients with jawbone destruction caused by tumors. Jawbone destruction was defined as a radiologically identifiable bone defect that was characterized by cortical discontinuity, whereas patients with only diffuse osteopenia without cortical disruption were excluded. This resulted in the selection of 383 patients with confirmed tumor-induced jawbone destruction. Sixty of them who underwent radiation therapy or chemotherapy were selected. Two patients were excluded because of previous administration of bone-modifying agents, resulting in a final study population of 58.

### Clinical factors

**Fig. 1:**
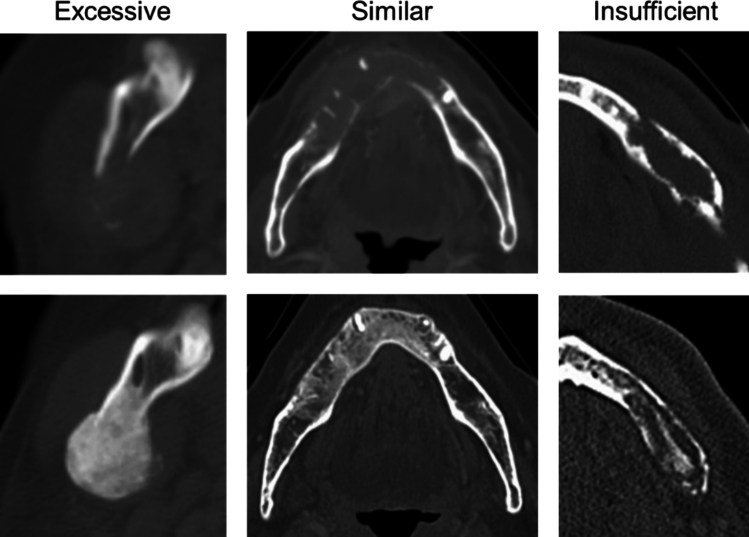
Categorization of jawbone morphology following remineralization. The remineralization morphology was classified into three categories: morphological overgrowth or outward bulging beyond the expected contour of the jawbone as “excessive” (left); closely matching the expected morphology as “similar” (middle); and incomplete morphology relative to the expected contour as “insufficient” (right)

The data of the patients were retrospectively analyzed to investigate the factors influencing bone remineralization and changes in CT values over time. The patient characteristics and clinical data extracted from the medical records included sex (female or male), age group (younger than 18 years or 18 years and older), tumor histology (carcinoma, hematopoietic tumors, or others, including sarcoma and neuroblastoma), primary site, site of jaw destruction (maxilla or mandible), type of bone destruction, and radiation dose to the jaw. Bone destruction was categorized as invasive (cortical bone discontinuity due to direct extension from adjacent tumors) or metastatic (bone destruction centered within the jawbone due to a mass lesion, where a primary tumor in another organ was confirmed). All hematopoietic tumors were considered to be involved in metastatic bone destruction. The radiation dose was categorized into three: no irradiation (only chemotherapy), less than 60 Gy (typically for palliative care or pre-transplant conditioning), and 60 Gy or more (intended for curative treatment of solid cancers). The jawbone destruction sites were assessed and classified into three categories based on the observed changes: remineralization, no significant change, and bone resorption. The jawbone morphology post-remineralization was evaluated against the anticipated normal morphology, which was defined as a standard based on the contralateral side, without consideration of bone density or trabecular pattern. In cases where contralateral comparison was not possible, such as in the anterior region, the anatomical morphology from radiological atlases was used as the reference. “Excessive” remineralization was defined as morphological overgrowth or outward bulging beyond the expected contour of the jawbone; cases that closely matched the expected morphology were categorized as “similar,” and those showing incomplete morphology relative to the expected contour were categorized as “insufficient” (Fig. 1). Remineralization was confirmed by two radiologists (K.S., with 7 years of experience, and M.K., with 16 years of experience).

### CT value measurement

CT values were measured for the patients with confirmed jawbone remineralization after follow-up for a minimum of 12 months from the initial confirmation of jawbone destruction (designated 0 months). Follow-up CT images were obtained at approximately 3, 6, and 12 months. Patients with missing data at any time point were excluded to ensure data integrity. Of the initial group of 23 patients with CT follow-up data accumulated for > 12 months, six patients were excluded because CT scans including the jawbone were not obtained at the study-defined time points; this was possibly owing to follow-up imaging being focused only on the primary tumor site or performed using other modalities such as MRI or panoramic radiography. Consequently, a final group of 17 patients was used for analysis. The mean times of the actual measurements were 0, 2.7, 6.2, and 12.1 months, with standard deviations of 0, 0.77, 1.3, and 1.7 months, respectively. CT values were measured for the trabecular bone at sites of bone destruction or remineralization. They were also measured for an anatomically comparable healthy region with no cancer-related changes, which served as the control.

**Fig. 2:**
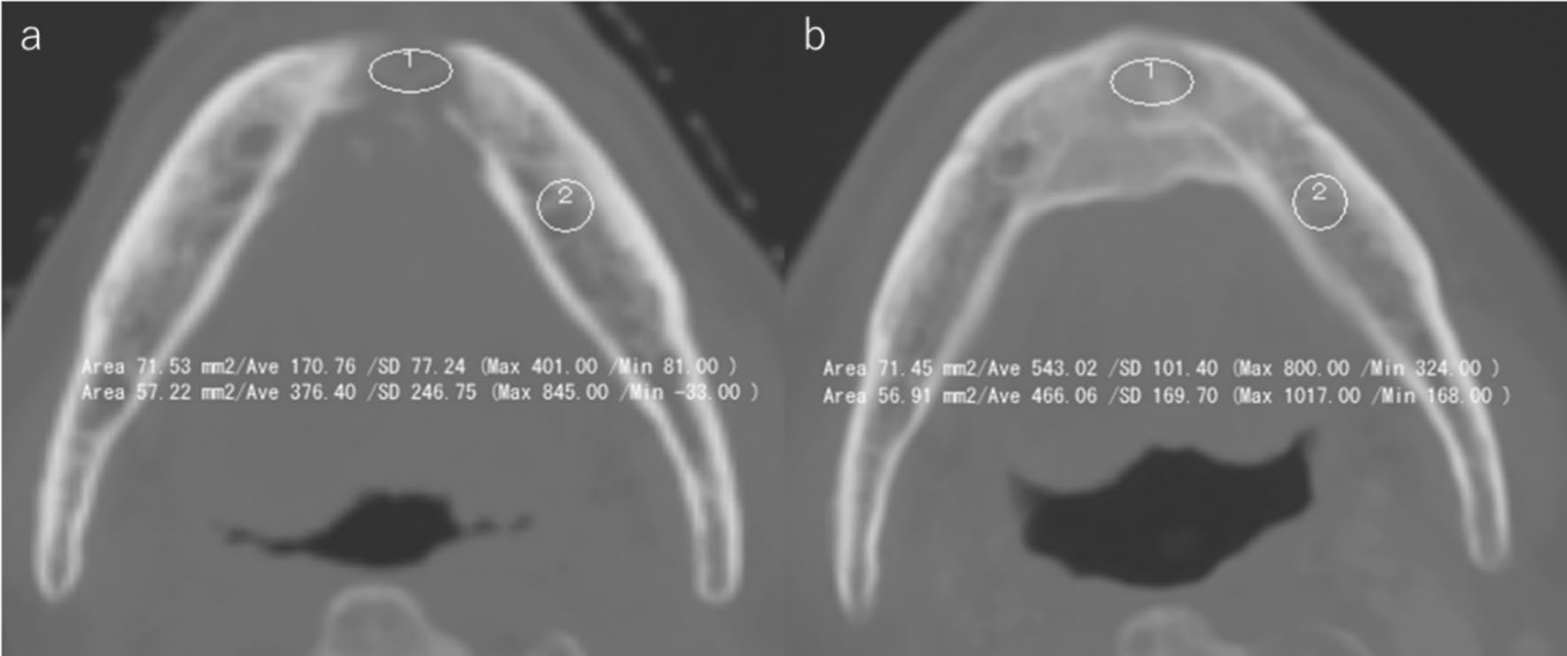
CT images at bone destruction **a** and remineralization **b**, with regions of interest (ROIs) placed for CT value measurement. ROIs were set within the trabecular bone at the destruction site and at the contralateral site or a molar region as reference. Follow-up images were selected at approximately the same level, with ROIs matched in size and location to those at baseline

CT values were measured using regions of interest (ROIs) located in the axial plane of the CT images. For each patient, the axial slice showing the most extensive bone destruction was selected; the ROIs were designed to be as large as possible in a quasi-circular shape within the trabecular bone at destruction site. The reference ROI was placed in the corresponding contralateral site when available or in the trabecular bone adjacent to a molar tooth if the contralateral site was not suitable. During follow-up, axial slices approximating the same anatomical position were selected; ROIs were placed to match the location and size of the initial ROIs, ensuring consistent paired comparisons between destruction, remineralization, and reference sites (Fig. 2).

### Positron emission tomography–computed tomography evaluation

We analyzed the PET/CT scans at three time points to explore the imaging features associated with bone formation: at the time of jawbone destruction (designated as 0 months); before remineralization during treatment; and after remineralization. The fluorodeoxyglucose F18 (^18^F-FDG) uptake and standardized uptake value maximum (SUVmax) values were recorded from the PET/CT scans. SUVmax was measured by placing an elliptical ROI covering the lesion site.

### Statistical analysis

All statistical analyses were performed using JMP Pro version 18.1.0 (SAS Institute Inc., Cary, NC, USA). Fisher’s exact test was used for 2 × 2 contingency tables with low expected counts [25], and the Chi-squared test was used for larger tables to determine statistically significant differences in bone remineralization associated with clinical factors. The relationships between clinical factors and jawbone morphology following remineralization were also evaluated using the Chi-squared test. Standardized residuals were calculated to assess the strength of association for each variable that showed statistically significant differences on Chi-squared tests. The Mann–Whitney *U* test was used to compare the mean CT values for the sites of bone destruction or remineralization and reference sites. The Friedman test was performed to assess changes in mean CT values over time at 0, 3, 6, and 12 months. A post hoc analysis with Bonferroni correction was performed to determine significant differences between specific time points. The Kruskal–Wallis test was used to assess the impact of clinical factors on the mean ratio of CT values at 12 months. Statistical significance was set at *p* < 0.05.

## Results

### Clinical factors related to bone remineralization

Of the 58 patients included in the study, 23 had bone remineralization, 25 showed no discernible changes, and 10 had progressive bone resorption. The progressive bone resorption group included four patients with exacerbated destruction and six patients with radiation-induced osteonecrosis of the jaw.

Clinical factors and outcomes of bone structure after nonsurgical treatment are summarized in Table 1. Statistical analysis revealed significant differences between the patients with remineralization and those with no significant changes (excluding bone destruction) in terms of age, tumor histology, site of jaw destruction, type of bone destruction, and radiation dose. Standardized residual analysis clarified which categories significantly contributed to remineralization status in the three-category cross-tables of tumor histology and radiation dose.

**Table 1:** Clinical factors and outcomes of bone structure after nonsurgical treatment

		Total *n* = 58	Remineralization *n* = 23	No significant changes *n* = 25	Bone resorption *n* = 10	P-value	Standardized residuals (remineralization/no significant changes)
Sex	Female	16	6 (38%)	7 (44%)	3 (19%)	1.000	
	Male	42	17 (40%)	18 (43%)	7 (17%)		
Age*	< 18 years	5	4 (80%)	0 (0%)	1 (20%)	0.0455 (Fisher)	
	≥ 18 years	53	19 (36%)	25 (47%)	9 (17%)		
Tumor histology*	Cancer	40	10 (25%)	21 (53%)	9 (23%)	0.0076	−2.932/2.932*
	Hematopoietic tumor	13	9 (69%)	4 (31%)	0 (0%)		1.801/−1.801
	Other	5	4 (80%)	0 (0%)	1 (20%)		2.178/−2.178*
Primary site	Oral cavity	19	5 (26%)	9 (47%)	5 (26%)	0.0593	
	Maxillary sinus	15	2 (13%)	9 (60%)	4 (27%)		
	Blood	13	9 (69%)	4 (31%)	0 (0%)		
	Lung	3	2 (67%)	1 (33%)	0 (0%)		
	Other	8	5 (63%)	2 (25%)	1 (13%)		
Site of jaw destruction*	Maxilla	25	5 (20%)	15 (60%)	5 (20%)	0.0095	
	Mandible	33	18 (55%)	10 (30%)	5 (15%)		
Type of bone destruction*	Invasive	35	7 (20%)	19 (54%)	9 (26%)	0.0033	
	Metastatic	23	16 (70%)	6 (26%)	1 (4%)		
Radiation dose*	Not irradiated	19	13 (68%)	6 (32%)	0 (0%)	0.0274	2.302/−2.302*
	< 60 Gy	9	4 (44%)	3 (33%)	2 (22%)		0.529/−0.529
	≥ 60 Gy	30	6 (20%)	16 (53%)	8 (27%)		−2.634/2.634*

^*^Statistically significant at *p* < 0.05

### Changes in CT values and associations with clinical factors

**Fig. 3:**
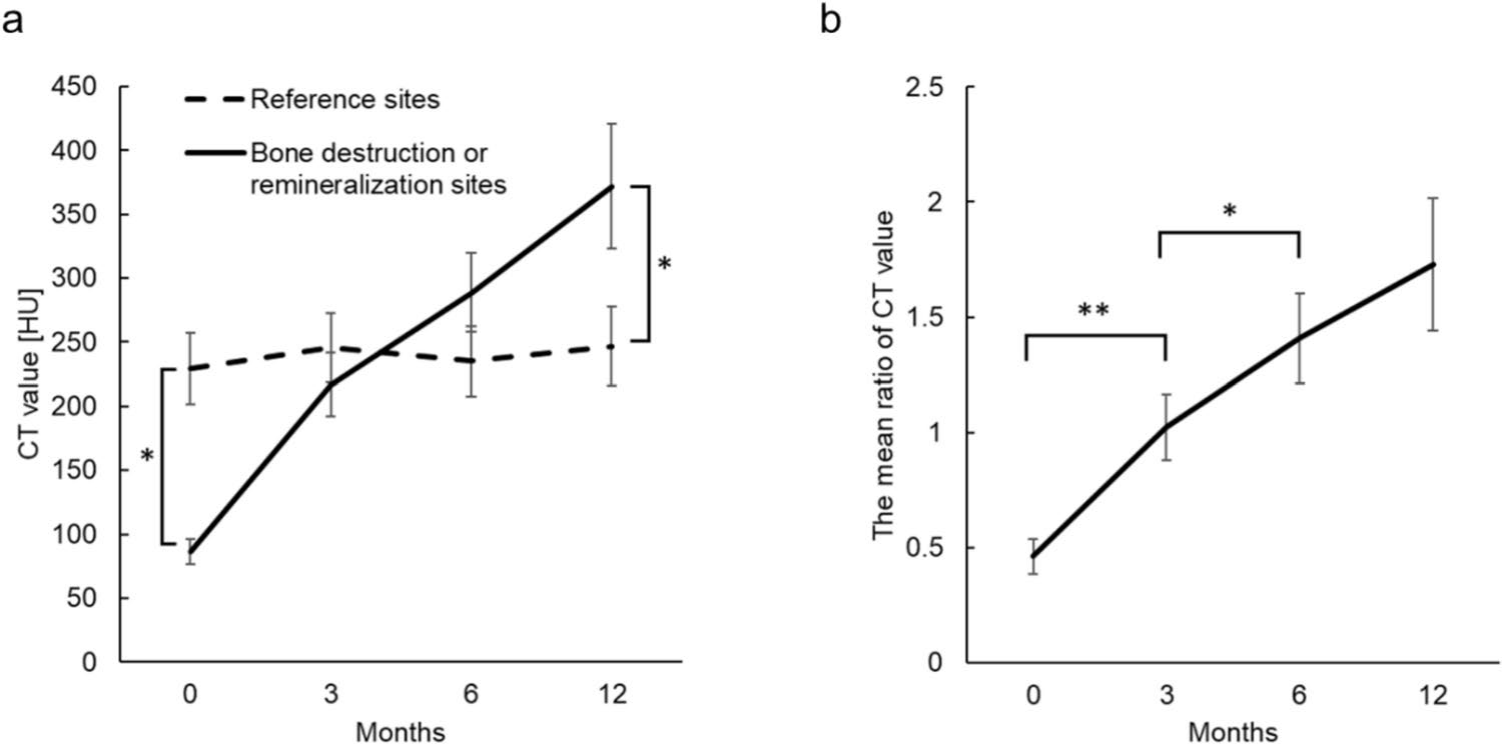
Line graphs of CT values at the bone destruction and remineralization sites at 0, 3, 6, and 12 months (**a**), and their ratios to the reference site (**b**) (*n* = 17). CT values at the reference site remained stable, whereas those at the destruction site increased with remineralization. The mean ratios of CT values (destruction or remineralization site divided by reference site) showed significant increases between 0–3 months and 3–6 months (*P-value < 0.05, **P-value < 0.01)

The mean CT values of the bone destruction sites at baseline (0 months) were significantly lower than those of the reference sites (Fig. 3a). The mean CT values of the remineralization sites at 12 months were significantly higher than those of the reference sites.

**Fig. 4:**
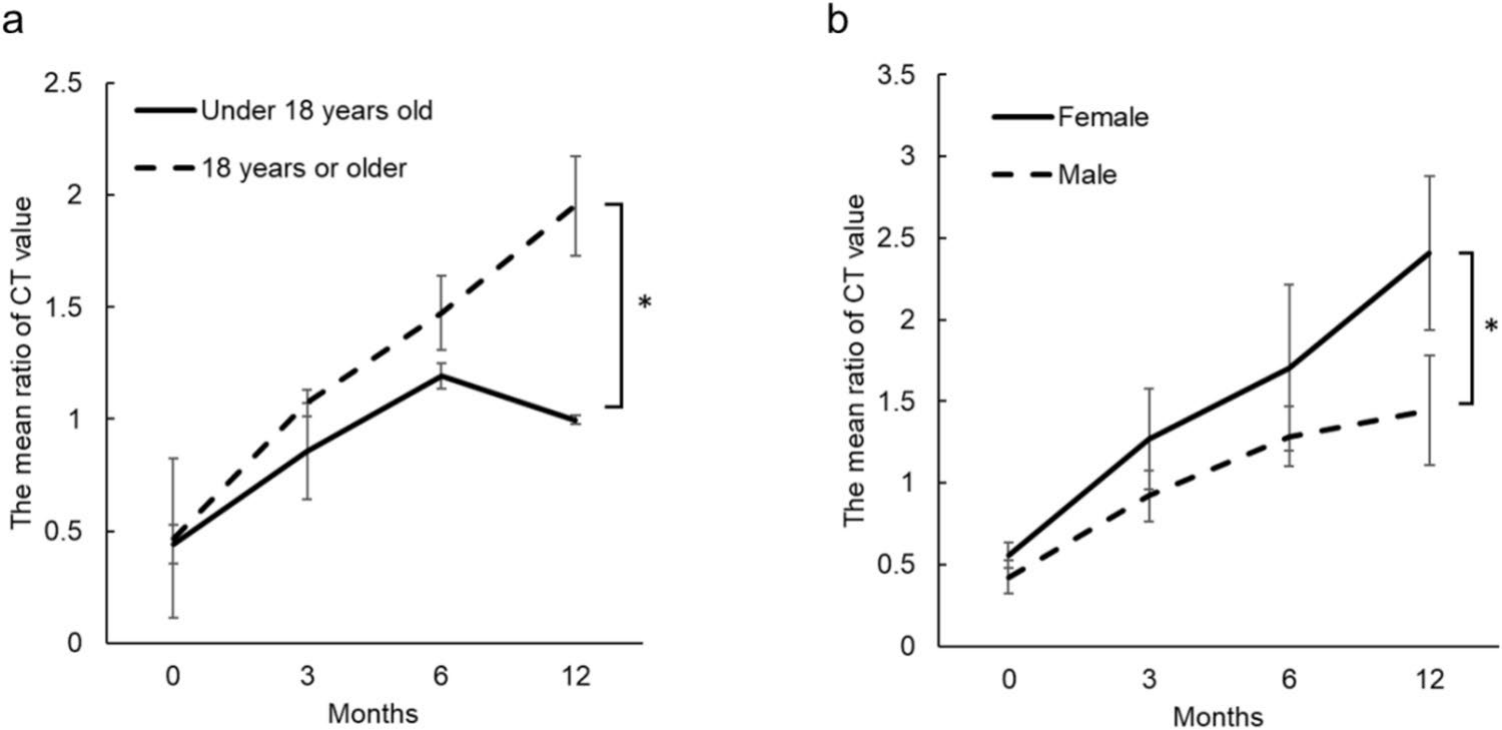
Line graphs of the ratios of CT values at the bone destruction and remineralization sites to the reference site at 0, 3, 6, and 12 months. Significant differences at 12 months were observed for age (**a**) and sex (**b**) groups. At 12 months, patients aged younger than 18 years (*n* = 4) had lower CT value ratios than those aged 18 years or older (*n* = 13), and males (*n* = 12) had lower ratios than females (*n* = 5)

The mean ratios of the CT values (destruction or remineralization sites to reference sites) increased progressively over the study period. The ratios were 0.46, 1.02 at 3 months, 1.41 at 0, 3, and 6 months, and 1.72 at 12 months, respectively (Fig. 3b). Statistically significant increases in the CT value ratios were observed between 0 and 3 months and between 3 and 6 months. Among the clinical factors, only age and sex showed significant differences in CT values at 12 months; specifically, patients aged younger than 18 years and male patients exhibited significantly lower CT value ratios (Fig. 4).

### Imaging evaluation of bone remineralization

**Fig. 5:**
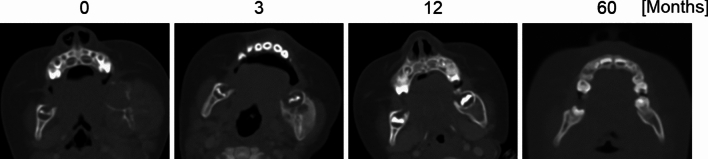
Representative CT images showing the transition from bone destruction to remineralization and bone remodeling in a 9-year-old patient. Bone destruction is observed in the left mandible at 0 months. Excessive remineralization was observed at 3 months, followed by bone remodeling at 12 months, resulting in a morphology approximating the anticipated normal contour at 60 months

The changes in the shape of the remineralized bone based on comparison with the anticipated normal are summarized in Table 2. Nine, 10, and 4 patients showed excessive, similar, and insufficient changes in the remineralized bone, respectively. No significant differences were identified between bone morphology and clinical factors. The morphology after initial remineralization was maintained throughout the study for 19 patients with no observable changes over time. However, subsequent imaging revealed remodeling toward normal bone morphology in four patients with excessive remineralization; three of these patients were aged younger than 18 years (Fig. 5).

**Table 2:** Clinical factors and changes in the morphology of remineralized jawbone relative to those of the reference site

		Total *n* = 23	Excessive *n* = 9	Similar *n* = 10	Insufficient *n* = 4	*P*-value
Sex	Female	6	1 (17%)	4 (67%)	1 (17%)	0.3582
	Male	17	8 (47%)	6 (35%)	3 (18%)	
Age	< 18 years	4	3 (75%)	1 (25%)	0 (0%)	0.2448
	≥ 18 years	19	6 (32%)	9 (47%)	4 (21%)	
Tumor histology	Cancer	10	5 (50%)	4 (40%)	1 (10%)	0.1717
	Hematopoietic tumor	9	1 (11%)	5 (56%)	3 (33%)	
	Other	4	3 (75%)	1 (25%)	0 (0%)	
Primary site	Oral cavity	5	2 (40%)	2 (40%)	1 (20%)	0.2095
	Maxillary sinus	2	2 (100%)	0 (0%)	0 (0%)	
	Blood	9	1 (11%)	5 (56%)	3 (33%)	
	Lung	2	2 (100%)	0 (0%)	0 (0%)	
	Other	5	2 (40%)	3 (60%)	0 (0%)	
Site of jaw destruction	Maxilla	5	3 (60%)	0 (0%)	2 (40%)	0.0683
	Mandible	18	6 (33%)	10 (56%)	2 (11%)	
Type of bone destruction	Invasive	7	3 (43%)	3 (43%)	1 (14%)	0.9548
	Metastatic	16	6 (38%)	7 (44%)	3 (19%)	
Radiation dose	Not irradiated	13	4 (31%)	7 (54%)	2 (15%)	0.8307
	< 60 Gy	4	2 (50%)	1 (25%)	1 (25%)	
	≥ 60 Gy	6	3 (50%)	2 (33%)	1 (17%)

**Fig. 6:**
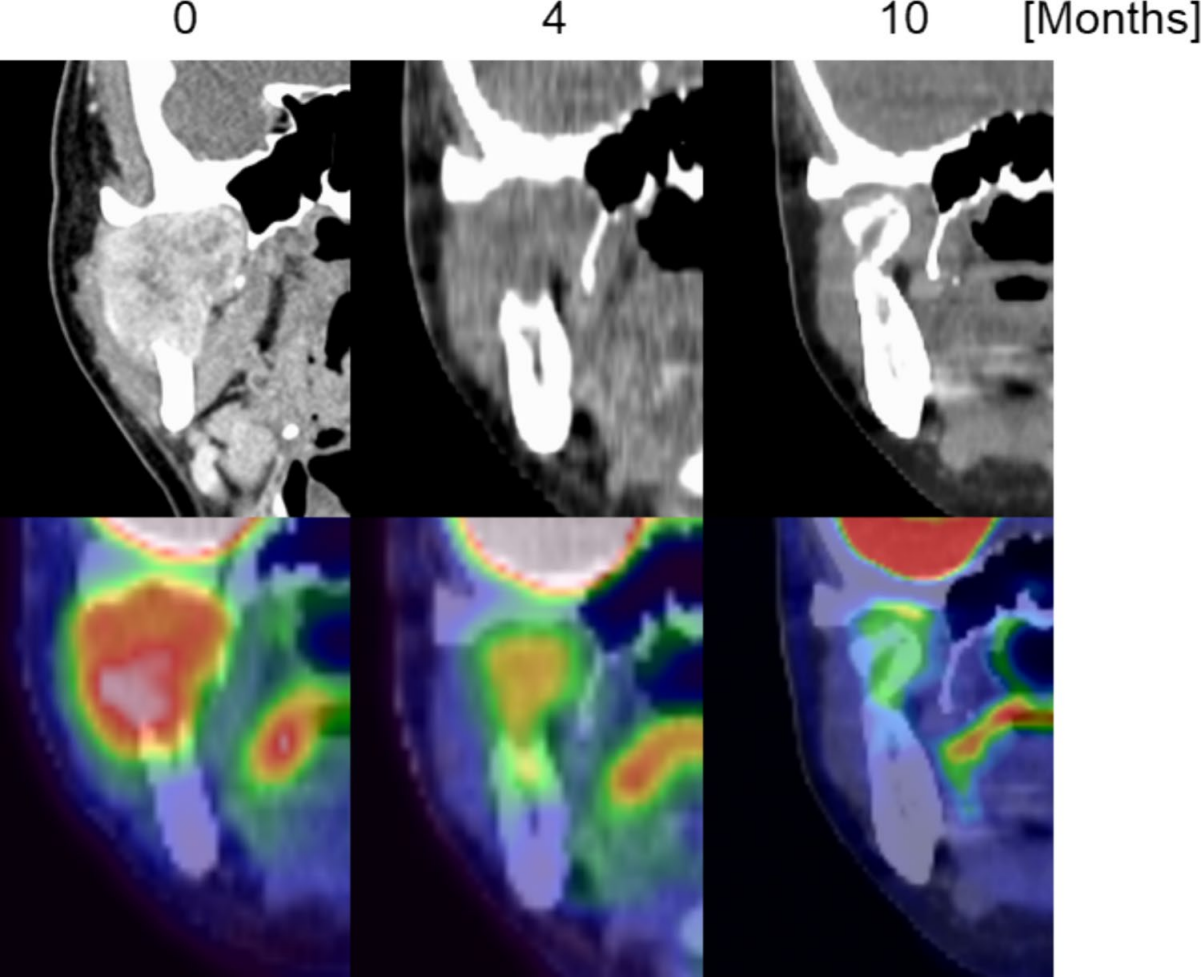
PET/CT images of a patient with bone metastasis to the right condyle at baseline (0 months), 4 months, and 10 months post-chemotherapy. At 0 months, abnormal fluorodeoxyglucose F18 (^18^F-FDG) standardized uptake value maximum (SUVmax = 4.7) was observed in the right condyle. At 4 months, CT did not show bone remineralization but ^18^F-FDG uptake persisted (SUVmax = 3.4). At 10 months, bone remineralization was visible at the same site with corresponding morphology

PET/CT images were examined for cellular metabolism during remineralization in two patients who underwent imaging during this period. The PET/CT findings for one patient are presented below as a representative case. At 0 months, PET/CT images showed bone metastasis to the right condyle due to renal cell carcinoma, with an ^18^F-FDG SUVmax of 4.7 (Fig. 6). The right condyle was not discernible on CT images at 4 months after chemotherapy initiation; however, the PET images revealed an accumulation of ^18^F-FDG with an SUVmax of 3.4. Bone remineralization was observed at 10 months, with a morphology corresponding to an area of high ^18^F-FDG accumulation at 4 months. The shapes and positions of the remineralized bone and the normal condyle were similar but not perfectly matched. The other patient showed a similar pattern with high ^18^F-FDG accumulation, followed by remineralization at the corresponding site.

## Discussion

This study demonstrated that remineralization occurred in approximately half of the patients with jawbone destruction following radiation therapy or chemotherapy, which is consistent with earlier observations in the vertebrae [12, 14, 20]. However, our research extended these previous findings by targeting the jawbone and including participants with a wider age range and varied tumor histology encompassing invasive and metastatic bone destruction. This wider focus facilitated the identification of additional factors influencing remineralization. Our findings revealed that age, tumor histology, site of jaw destruction and type of bone destruction were significantly associated with remineralization. These add to sex, tumor histology, and radiation dose, which have been reported in previous studies [13–19].

Several previous studies have reported a significant association between the type of primary tumor and bone remineralization [12–14, 20]. Our study found a similar trend for the tumor primary site, but this association was not statistically significant. A statistically significant difference was observed between tumor histology and remineralization, suggesting that histology-specific factors may influence remineralization. In addition, the pattern of bone destruction—classified as either invasive or metastatic—was also significantly associated with remineralization. These results support the possibility that the factors promoting remineralization are inherent to the tumor itself and not its original location.

Reports regarding the relationship between radiation and remineralization have been inconsistent; some studies have reported a significant association [12, 20, 22] while others have not detected such a correlation [14, 26, 27]. The radiation dose applied to the vertebrae in those studies ranged from 8 to 40 Gy, corresponding to the less than 60 Gy category in our study. No significant difference in the remineralization of the jawbone was detected for this category.

Further, our study suggested that remineralization occurred more frequently in patients who had not undergone radiation therapy than in those who had. The group without radiation therapy received chemotherapy alone, and remineralization was observed even in the absence of radiation exposure. Chemotherapy agents and radiation dose have diverse effects on bone metabolism and the inflammatory cascade, and their precise impact on remineralization remains incompletely understood [28, 29]. Furthermore, factors derived from tumor cell death may contribute to the induction of bone formation [30], suggesting that bone regeneration is regulated through the complex interplay between therapeutic modalities, tumor cells, and normal cellular responses.

CT values have been widely used to measure bone mineral density at remineralized sites [22]. However, bone mineral density varies within the jawbone depending on the anatomical location and specific jaw regions. To account for this variability, this study employed the mean ratio of CT values at the bone destruction or remineralization sites to those at the reference sites for comparison with clinical factors.

Previous research has reported that bone remineralization is often evident approximately 3 months after treatment [31], with subsequent CT values at remineralized sites exceeding those at reference sites [12]. We confirmed remineralization at approximately 3 months, with the CT value ratio of the remineralized sites to the reference sites reaching 1.02. The CT value ratio at the remineralized sites markedly increased to exceed the reference site value of 1.7 by 12 months.

Our study also demonstrated that patient age and sex significantly influenced the 12-month CT value ratios. Patients younger than 18 years and male patients presented with significantly lower CT values at the remineralized sites. Previous studies did not include this age group, making this the first study to establish that age plays a role in remineralization, although the number of patients younger than 18 years was limited. Some reports have shown no significant association between sex and remineralization [20, 26]. These reports included patients taking bone-modifying agents, which may have confounded earlier results. From a biological rationale, delayed bone remodeling due to cellular senescence as well as hormonal differences between sexes may account for the observed changes in CT values [32, 33]. These factors suggest that physiological bone metabolism and growth-related processes should be considered when interpreting remineralization outcomes.

Bone-modifying agents, such as bisphosphonates, affect CT values [13, 14, 20]. These agents are commonly prescribed to prevent fractures in patients with osteoporosis and bone metastases in cancers such as breast cancer, where bone metastases frequently occur [6, 8–10]. However, these medications carry the risk of medication-related osteonecrosis of the jaw, which can influence bone remineralization after metastasis. To eliminate this potential confounder, our study excluded patients taking bone-modifying agents. This may also explain the relatively few patients with breast cancer in our cohort.

Previous research has suggested that the primary tumor type may influence CT values after remineralization [14]. However, our study did not find statistically significant associations between the primary tumor type and CT values. Unlike earlier investigations that focused solely on bone metastases, our study analyzed metastatic bone destruction and invasion of tumors adjacent to the jawbone. This difference may have contributed to the significant differences observed.

The morphology of the remineralized jawbone is remarkably varied, echoing the diversity observed in vertebral remineralization [17, 18]. We classified remineralization morphologies as excessive, similar, or insufficient compared with the reference sites and found no associations with clinical factors. The initial morphology after remineralization persisted over time for most cases, and no changes were detected. However, some patients, especially those younger than 18 years, initially had excessive remineralization. This was followed by a shift toward normal bone morphology indicating active bone-remodeling processes. Our study revealed that most patients retained their remineralization morphology for a few years, whereas those who eventually regained their original bone shape required significantly longer than is typical for fracture healing. This prolonged process may be influenced by various factors, such as the size of the bone destruction site, presence of tumor cells, and effects of radiation and chemotherapy [29, 34].

PET/CT images further enriched our findings, capturing metabolic activity associated with bone remineralization. These findings suggest that bone remineralization occurs as a localized mass of bone formation, which is unlike the gradual healing processes observed during cyst removal. In addition, previous studies have reported that remineralization occurs through the calcification and ossification of collagen fibers aggregating within fibrous tissue that replaces dead metastatic cancer cells after irradiation; this remineralization is thought to correspond to the callus formation process during fracture healing [35]. The ^18^F-FDG accumulation observed at remineralized sites in this study supports the notion that the mechanism of bone remineralization may be similar to that of fracture healing, consistent with previous reports of ^18^F-FDG accumulation at fracture sites on PET/CT images [36, 37]. It should be emphasized that ^18^F-FDG reflects metabolic activity rather than bone formation itself [38]; only two patients in our cohort underwent PET/CT during the remineralization period, which limits the strength of interpretation of the study results. Nonetheless, the ability to visualize localized metabolic activity preceding morphological remineralization provides valuable insight into the cellular processes that may shape the eventual bone morphology; we believe this observation warrants further investigation.

This study had several limitations. These include the relatively small sample size, as the study was conducted at a single institution. A multicenter collaboration is recommended to increase the sample size, given the low incidence of jawbone metastases (1–8% of all bone metastases) [6, 8]. Furthermore, the retrospective nature of the study introduced variability in CT scans, as they were performed using different protocols and equipment across multiple facilities [39]. Nonetheless, the use of reference sites likely mitigated the impact of this variability.

In summary, this study provides valuable clinical insights into the factors influencing jawbone remineralization after tumor-induced destruction. We demonstrate that age, sex, tumor histology, the site and type of bone destruction, and radiation dose affect the occurrence and morphology of remineralization. Recognizing these factors may help clinicians to predict the course of bone healing and to optimize jawbone surveillance in individual patients. Furthermore, the observation of metabolic activity corresponding to remineralization on PET/CT may support recovery monitoring and inform decisions on post-treatment interventions, including the necessity and timing of surgical reconstruction or prosthetic rehabilitation. Overall, these findings may contribute to treatment planning and enhance clinical decision-making for patients with jawbone destruction.

## Data Availability

The data that support the findings of this study are not openly available due to reasons of privacy concerns and are available from the corresponding author upon reasonable request.
